# Implementation Plan for a High-Frequency, Low-Touch Care Model at Specialized Type 1 Diabetes Clinics: Model Development

**DOI:** 10.2196/37715

**Published:** 2022-12-08

**Authors:** Stephanie de Sequeira, Justin Presseau, Gillian L Booth, Lorraine L Lipscombe, Isabelle Perkins, Bruce A Perkins, Rayzel Shulman, Gurpreet Lakhanpal, Noah Ivers

**Affiliations:** 1 Unity Health Toronto Toronto, ON Canada; 2 Ottawa Hospital Research Institute Ottawa, ON Canada; 3 Women's College Hospital Toronto, ON Canada; 4 Sinai Health System Toronto, ON Canada; 5 The Hospital for Sick Children Toronto, ON Canada

**Keywords:** type 1 diabetes, virtual care, high-frequency care, implementation science, diabetes, support, incentives, clinics, intervention, behavior change, education, glycemic control, self-management

## Abstract

**Background:**

Individuals with type 1 diabetes (T1D) are more likely to achieve optimal glycemic management when they have frequent visits with their health care team. There is a potential benefit of frequent, telemedicine interventions as an effective strategy to lower hemoglobin A1c (HbA1c).

**Objective:**

The objective is this study was to understand the provider- and system-level factors affecting the successful implementation of a virtual care intervention in type 1 diabetes (T1D) clinics.

**Methods:**

Semistructured interviews were conducted with managers and certified diabetes educators (CDEs) at diabetes clinics across Southern Ontario before the COVID-19 pandemic. Deductive analysis was carried out using the Theoretical Domains Framework, followed by mapping to behavior change techniques to inform potential implementation strategies for high-frequency virtual care for T1D.

**Results:**

There was considerable intention to deliver high-frequency virtual care to patients with T1D. Participants believed that this model of care could lead to improved patient outcomes and engagement but would likely increase the workload of CDEs. Some felt there were insufficient resources at their site to enable them to participate in the program. Member checking conducted during the pandemic revealed that clinics and staff had already developed strategies to overcome resource barriers to the adoption of virtual care during the pandemic.

**Conclusions:**

Existing enablers for high-frequency virtual care for T1D can be leveraged, and barriers can be overcome with targeted clinical incentives and support.

## Introduction

Individuals with type 1 diabetes (T1D) are more likely to achieve optimal glycemic management when they have frequent visits with their health care team [1]. Further, prior clinical trials suggest a potential benefit of frequent telemedicine interventions as an effective strategy to lower hemoglobin A1c (HbA1c) among individuals with T1D [2,3]. However, it is not just the frequency of visits that has a significant effect on clinical and quality of life outcomes but also the type of interaction. There is a growing body of research on the effect of synchronous—or real-time—interactions (ie, in-person, phone calls, or video visits) compared to asynchronous interactions (ie, email or text). For example, Verhoeven et al [4] showed that synchronous telemedicine interactions lowered costs for both patients and the health care system by reducing unscheduled visits compared to usual in-person care. A model of care that includes frequent synchronous interactions between individuals with T1D and their health care teams is particularly beneficial to patients who are not meeting glycemic targets and need to make changes to their diabetes self-management [5]. Unfortunately, this was difficult to deliver in the context of pre-COVID-19 care, which typically involved time-consuming in-person visits during working hours. The necessary move to virtual care during the COVID-19 pandemic provided a window of opportunity to address this gap in T1D management through virtual models of care.

The T1ME (Type 1 Diabetes Virtual Self-Management Education and Support) trial aims to test the effectiveness of a model of high-frequency, low-touch (ie, virtual, remote) care with real-time visits for individuals with T1D who are not meeting glycemic targets (HbA1c >8%). If the T1ME trial is to be successful during the COVID-19 pandemic and beyond, it must be implemented using evidence-based processes [6]. Evidence-based implementation approaches help bridge the gap between the care that practitioners know is effective and that which is delivered [7] by understanding and targeting the contextual factors of the health care setting [6,8]. These complex contextual factors include organizational support [9], willingness of staff to participate in the intervention [10], and current health care delivery systems [11]. Implementation strategies must be developed to effectively target all these contextual factors. Additionally, most published multifaceted implementation strategies do not provide an explanation for why certain components were chosen [12], making it difficult to assess whether interventions sufficiently address known barriers.

Therefore, in this study, we sought to clarify the complex provider and system factors that need to be considered when implementing a high-frequency virtual care model in T1D diabetes clinics prior to and during the COVID-19 pandemic. Additionally, we sought to comprehensively describe how we developed an implementation plan suited to address the identified factors.

## Methods

### Study Design

This was a theory-informed, qualitative study seeking to understand the determinants of engagement with high-frequency virtual care in general and the T1ME trial components and to map those determinants to feasible implementation strategies.

### Ethics Approval

Ethics approval for this study was granted by the Women’s College Hospital (2018-0108-E) and the Ottawa Health Science Network Research Ethics Board (20190527-01H).

### Context

Clinical practice guidelines suggest that people with T1D have visits with their diabetes team every 3 months unless their glycemic management is already optimized [13]. More frequent visits with certified diabetes educators (CDEs) and other care providers are often needed to help patients recognize glucose patterns, adjust their insulin doses, and offer education and technical support on the use of insulin pumps and continuous or flash glucose monitoring. In Ontario, people with T1D may be eligible to receive government funding for insulin pumps and related supplies through Ontario Health’s Assistive Devices Program (ADP). Individuals registered in this program are required to receive frequent care from a certified pump team. Within this model of care, patients may need to wait 3 to 6 months for appointments with their diabetes team to troubleshoot issues with diabetes self-management. Prior to the COVID-19 pandemic, most visits were conducted in person, requiring individuals with T1D to take time off work or school to visit their team members. This model of care may not be well suited to patients who need additional support or timely enough to enable them to make real-time changes to their diabetes self-management.

The traditional T1D care model, which featured mainly in-person care, was applicable up until the pandemic [14]. In March 2020, diabetes clinics in Ontario, Canada, were mandated to adopt a virtual care model rapidly and with minimal preparation due to COVID-19 lockdown measures implemented. As of December 2021, most T1D care continues to be delivered virtually. However, despite virtualization, indicators suggest that care is still provided with longer, infrequent appointments every 3 to 6 months. Although second vaccination rates have surpassed 80%, and booster doses have surpassed 30% in Ontario [15], it is unlikely that T1D care will return to the prepandemic norm, especially with new variants arising. Instead, diabetes clinics will most likely adopt a “new normal” model of care that will include virtual options when in-person visits are not feasible or needed, as there will be lingering concerns regarding social distancing for some time, and many clinics have already invested in virtual care technologies. Virtualization of diabetes care offers an opportunity to consider shorter, more frequent contacts through more feasible virtual modalities.

The T1ME trial seeks to improve this T1D care model and focus on more patient-centered care, which will allow patients to become an important part of the care team and decision-making process. The T1ME trial is comprised of 3 components aimed at supporting self-management changes and goal advancement: (1) virtual care software that enables video (or audio and instant messaging) visits between patients and their health care providers; (2) automatic appointment reminders and goal setting prompts; and (3) a centralized virtual library that houses curated and vetted education and self-management resources for individuals living with T1D.

If our high-frequency virtual model is to be successful, we must target key workflow processes and behaviors among diabetes clinic staff. First, many traditionally in-person visits will need to be changed to virtual. This includes understanding and targeting workflow processes related to the uptake of new telecommunication technology. Second, we must understand the behavior changes in CDEs, clinic leaders and managers, and clinic support staff needed to accommodate a high-frequency care model. Within this model, patients will meet with their CDEs for shorter but more frequent touch points. This will change the nature of the interaction and affect workflow processes. Additionally, we will need to evaluate current resource allocation and the potential impact of our high-frequency, low-touch model on clinic resources. Therefore, in this study, we sought to understand workflow processes, resource allocation, and other factors in implementing a high-frequency, low-touch care model in diabetes clinics.

### Participants and Recruitment

We aimed to recruit between 30 and 40 nurses or dietician CDEs and managers in diabetes education programs at specialized T1D clinics in Southern Ontario. Sites were purposefully selected for variation in factors thought to potentially affect implementation of the intervention, including the total number of patients, number of patients under age 25, number of health professionals, number of patients with most recent HbA1c above 8%, and rurality. For each site, a recruitment email was sent to the lead physician or clinic manager inviting them to participate in a 30- to 45-minute telephone interview. We also sent invitation emails to CDEs and managers identified by the investigators’ personal networks. We then recruited additional key informants at each site using snowball sampling. In particular, we sought a team member of the chosen T1D clinic who provided clinical care or support and/or had knowledge regarding the organization of the clinic processes, including technological processes (eg, electronic medical records).

### Data Collection

First, an electronic survey was sent to the clinic manager at each clinic to obtain descriptive information about the clinic, including the number and type of health care professionals, types of communication with patients, wait times, and history with implementations of new programming. Author SdS then conducted semistructured 1-on-1 telephone interviews that were 30 to 45 minutes in duration during working hours. Interviews were recorded, deidentified, and transcribed. Oral informed consent was obtained before beginning the interviews. Field notes were made after each interview.

Interviews followed a semistructured guide (developed by authors NI, JP, SdS, GB, and LLL) that aimed to (1) explore current processes and procedures for management of T1D patients under routine and semiurgent scenarios and (2) examine the determinants of uptake and implementation of our high-frequency, low-touch model of care using the Theoretical Domains Framework (TDF). The TDF is an integrated framework synthesized from 128 theoretical constructs from 33 theories judged most relevant to implementation questions. Domains of the TDF include items such as knowledge, goals, optimizing, and belief about capabilities [16].

### Analysis

Research team members with a range of disciplinary backgrounds in endocrinology (GB), psychology (JP), family medicine (NI), and public health (SdS) reviewed the electronic survey data and transcriptions in depth to understand the current processes in the clinic and, importantly, the changes required for the intervention to be implemented as intended. The transcripts were examined to explore how the changes required might vary across clinics [17].

Transcriptions of the interviews were then coded using the TDF domains by 2 independent researchers (authors SdS and IP) using a word processor. Coding was mainly deductive, involving content analysis [18] and assigning utterances to the relevant TDF domains. Open coding was used when important issues were identified that did not seem to fit any existing domain. A codebook was maintained and updated regularly to ensure intercoder reliability.

When all transcripts were coded, authors NI, JP, SdS, and GB identified the most important determinants (domains) to be addressed in the implementation and training plan by (1) frequency (ie, which domains, and for which key targeted behavior, most commonly arise as issues to be addressed in the transcripts); (2) conflict (ie, presence of disagreement across participants on certain domains representing a potential need for tailored strategies); (3) strongly held and strongly emphasized beliefs amongst participants about the targeted behavior; and (4) most important determinants to be addressed (ie, determinants that have the highest likelihood of impeding or facilitating implementation) [19]. Additionally, themes within each domain were inductively coded into higher-level barriers and enablers. Then, we mapped out how each domain interacted with other domains. This allowed us to generate a list of theoretical domains most likely to influence the targeted behaviors for the successful implementation of the T1ME trial.

Finally, authors NI, JP, and SdS used the Behavior Change Techniques Taxonomy Version 1 (BCTTv1), developed through an international consensus process, to identify actions that would enable the interventions to become more easily adopted into routine care [20,21]. This taxonomy provides clarity surrounding the specific, active ingredients needed to elicit behavior change and draws on applied research in behavioral medicine, as well as social and health psychology. BCTs likely to influence key TDF domains have been previously mapped [21]. Team members (authors NI and JP) with training and experience identified the most promising BCTs thought to be feasible to utilize in the implementation and training strategies for the intervention. We used these BCTs and the most relevant theoretical domains to create a comprehensive implementation and training plan, which could be tailored to each site if necessary.

Analysis and data collection occurred concurrently, and recruitment ceased once thematic saturation was reached. Our threshold for thematic saturation was 2-fold. First, our initial analysis sample (minimum sample size) included at least 1 CDE and 1 manager from each site. Following that, our stopping criterion was a 0% new information threshold in the key theoretical domains [22,23].

### Member-Checking Calls

Author SdS conducted member-checking calls with participants to ensure that our interpretation of the barriers and enablers from the original interviews accurately reflected the context of their specific clinics [24]. Since the member-checking calls were conducted during the COVID-19 pandemic, we also took the opportunity to inquire about whether our interview results held true during the context of completely virtual care and understand processes that clinics initiated to accommodate virtual care.

Member-checking calls were recorded, deidentified, and transcribed. Field notes were made during the member-checking call. Two independent researchers (authors SdS and IP) coded the transcripts using a word processor. We used deductive analysis, assigning quotes to the barriers and enablers from the original interviews. We also used open coding for issues other than the barriers and enablers identified in the interviews. A member-checking codebook was maintained and updated regularly to ensure intercoder reliability.

## Results

### Participants and Sites

Between February 1 and May 16, 2019, we interviewed 35 participants across 12 diabetes clinics in Southern Ontario. Of the 35 interviews completed, 20 (57%) participants were CDEs, 13 (37%) were managers, 1 (3%) was an administrative coordinator, and 1 (3%) was a social worker. Two sites declined participation, and 3 sites did not respond to the invitation email. Table 1 contains the demographic information and process in each participating clinic.

**Table 1:** Descriptive information about Ontario Health’s Assistive Devices Program (ADP) pump sites.

Clinic	Providers per site or network^a^, n	EMR^b^	Minutes per day calling or emailing patients^c^	Methods used for virtual communication (other than in-person visits)	
				Prepandemic	Postpandemic
1	23	Yes	20-40	Telephone, email	N/A^d^
2	81	Yes	>60	Telephone, email	Telephone, email, Zoom
3	25	Yes	>60	Telephone, email, OTN^e^	Telephone, email, Zoom
4	16	No	>60	Telephone, email, OTN	Telephone, email
5	22	Yes	40-60	Telephone, email	Telephone, email, Zoom
6	28	Yes	>60	Telephone, email	Telephone, email, WebEx
7	15	No	20-40	Telephone, email, OTN	Telephone, email
8	8	Yes	N/A	Telephone, email, SMS	Telephone, email, Zoom
9	19	Yes	20-40	Telephone, email	Telephone, email, OTN, MS^f^ teams
10	15	Yes	40-60	Telephone, email, OTN, SMS	Telephone, email, OTN, Zoom
11	15	Yes	N/A	N/A	Telephone, email, Zoom
12	N/A	No	N/A	N/A	N/A

^a^All health care providers for type 1 diabetes (T1D) care (endocrinologists, nurses, dieticians, etc); based on full-time equivalent, rounded-up.

^b^EMR: electronic medical record.

^c^Prepandemic.

^d^N/A: not applicable.

^e^OTN: Ontario Telemedicine Network.

^f^MS: Microsoft.

### Key Barriers and Enablers to High-Frequency, Low-Touch Care

We separated our findings into the 2 components of the T1ME trial (ie, high frequency and low touch).

Within the component of low-touch (virtual) care, we found 2 main barriers. First, there was the belief that low-touch care would lead to an increased workload. This included double administrative work and increased time and work spent on troubleshooting technical glitches. As 1 participant stated, “*It takes up a very [large] amount of our health care practitioners’ time to troubleshoot the technology”* [participant #19].

Second, managers reported that there was a lack of financial resources to obtain virtual care technology and a lack of private clinic space and offices to offer virtual care.

Alternatively, participants noted that there was an interest and intention to use virtual care. However, this interest and intention varied depending on the CDE, manager, and institution. For example, as 1 CDE noted, their “*organization as a whole wants [low-touch care], and [I] know that part of their strategic direction for the next five years is to increase virtual visits, so this aligns with that*” [#15]. On the other hand, some managers wanted to observe the success of the program before agreeing to participate: “*If the feedback is positive, then yes, absolutely*” [#22]. Finally, the CDEs and managers who believed that low-touch care would improve patient outcomes and had existing skills and comfort with virtual care (ie, phone, email, video visits) had more of an intention to participate in the T1ME trial.

We also found barriers and enablers relating to high-frequency care. First, the belief that high-frequency care would increase the workload of charting and documentation was a barrier to the uptake of the T1ME trial. Second, clinic staff reported that there was a lack of resources such as staff, capacity, and time to successfully implement high-frequency care. For example, 1 manager stated that she was *“very hesitant about [participating in the T1ME trial], just because the volumes that we deal with and the admin support that we have, we just can’t handle that*” [#6].

However, clinic staff who believed that high-frequency care would lead to better patient outcomes and increased patient engagement were more likely to participate in the T1ME trial. As 1 CDE stated, high-frequency care would be “wonderful” for patients:

More frequent low-touch follow-up is probably going to be a wonderful thing for them, a way to check in or get questions clarified. Now, I’m trying this out. Now, the rubber meets the road. Here’s a little hiccup. Being able to troubleshoot that.

#27

To further enable the successful implementation of the T1ME trial as a whole, engagement of CDEs throughout the T1ME trial was deemed necessary:

Most of the staff here are very open to trying different things if the patients want it. Again, it has to be something that the patients are willing to do.

#5

Positive patient feedback, adoption of the program by a local champion and other clinic staff, and continuing support from the T1ME trial team were suggested as methods to encourage CDE participation and offer ongoing engagement in the program.

### Theoretical Domains

We identified that themes coded within some theoretical domains related to those in the same domain and other domains. For example, there were 3 themes within the “beliefs about consequences” domain: (1) improved patient engagement, (2) improved patient outcomes, and (3) increased workload. If participants believed that the T1ME trial would increase their workload, they reported less intention to participate. On the other hand, if participants believed that the T1ME trial would improve patient outcomes and engagement, they were more likely to participate in the program. Therefore, multiple different beliefs about consequences likely affected the relative intention to participate.

**Figure 1:**
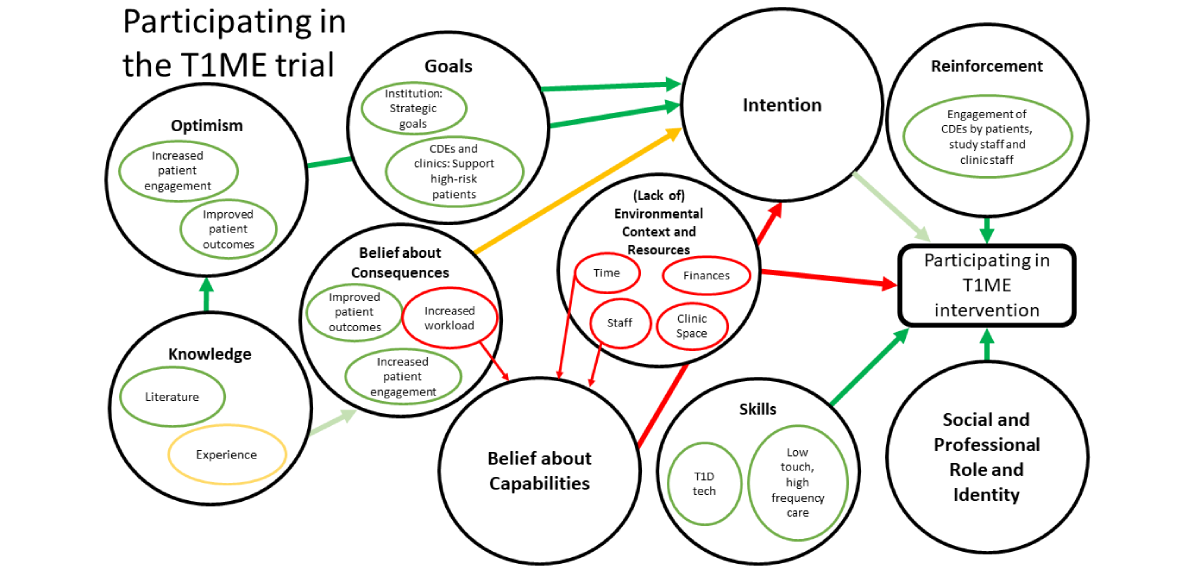
Map of interactions between theoretical domains. Large black circles show Theoretical Domains Framework (TDF) domains important to our project. Smaller circles within the large circles identify concepts that were identified during our analysis. Green arrows and circles indicate facilitators (the darker the green, the stronger the facilitator). Yellow arrows and circles depict a mixed effect. Red arrows and circles show the barriers.

**Figure 2:**
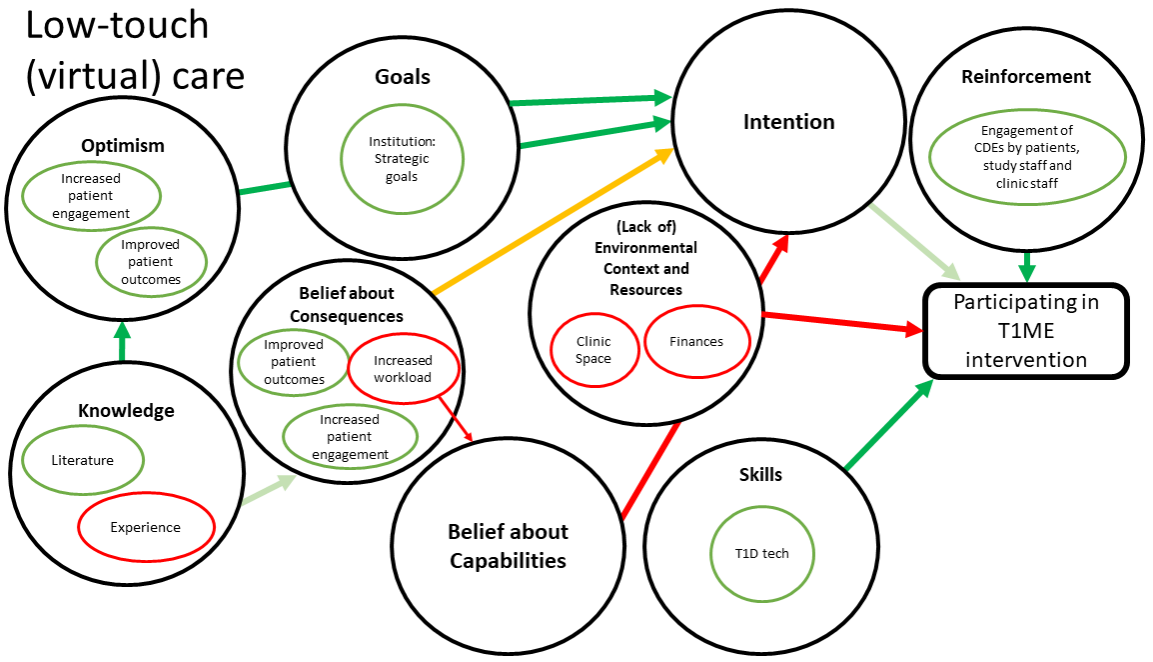
Map of interactions between theoretical domains for low-touch (virtual) care. Large black circles show Theoretical Domains Framework (TDF) domains important to our project. Smaller circles within the large circles identify concepts that were identified during our analysis. Green arrows and circles indicate facilitators (the darker the green, the stronger the facilitator). Yellow arrows and circles depict a mixed effect. Red arrows and circles show the barriers.

**Figure 3:**
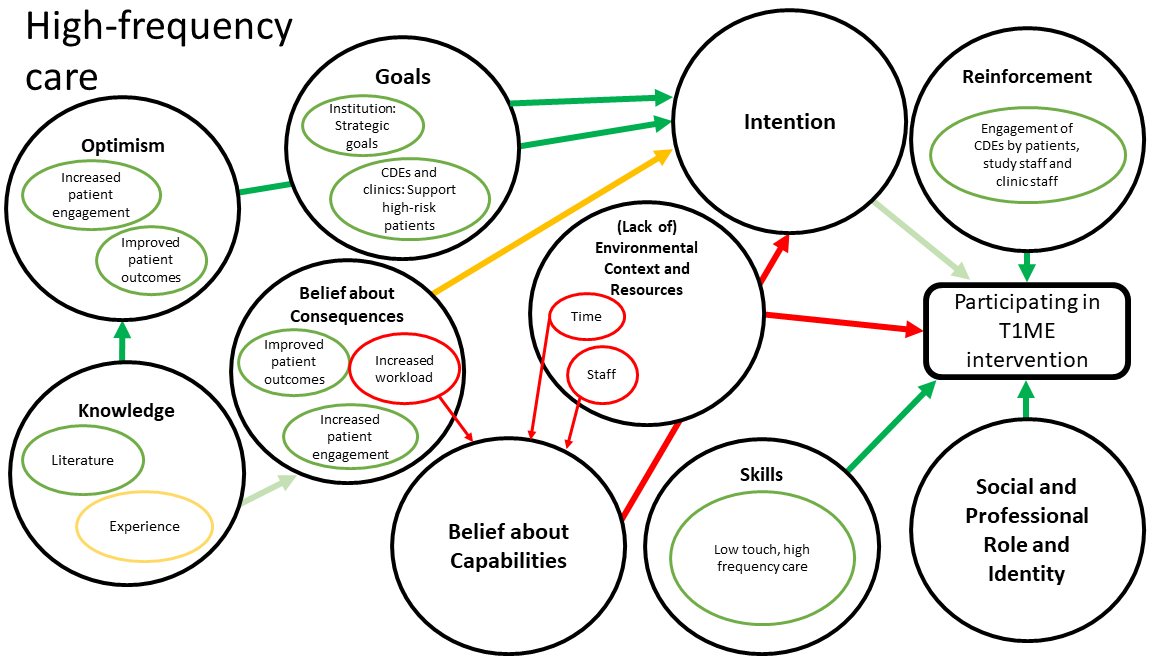
Map of interactions between theoretical domains for high-frequency care. Large black circles show Theoretical Domains Framework (TDF) domains important to our project. Smaller circles within the large circles identify concepts that were identified during our analysis. Green arrows and circles indicate facilitators (the darker the green, the stronger the facilitator). Yellow arrows and circles depict a mixed effect. Red arrows and circles show the barriers.

Figure 1 maps how constructs within domains may relate to other domains and ultimately affect clinic staff participation in the T1ME trial. Figure 2 exhibits the map for low-touch care, which contains the theoretical domains of knowledge, optimism, belief about consequences, goals, skills, reinforcement, and intention. In addition to the aforementioned domains, the high-frequency component (Figure 3) was informed by the domain of social and professional roles and identity.

### Member-Checking Calls

Between June 23 and September 10, 2020, we completed member-checking calls with 20 of our 35 participants at 8 out of 12 sites. We spoke to 13 CDEs, 6 managers, and 1 administrative coordinator. Some of the staff we originally interviewed were redeployed to COVID-19 testing centers at their respective institutions and were therefore unable to complete the member-checking call.

In general, participants agreed with the barriers and enablers we previously identified in this paper. Additionally, a few opportunities arose during the COVID-19 pandemic. First, even though participants agreed with the barriers, 1 CDE noted that barriers never impeded offering care to patients who needed it:

I would say that staffing has always been an issue to some extent…but I don’t know that it necessarily affected our ability to see those patients who really needed to be seen.

#16

Additionally, some clinics invested in technology, such as laptops for staff to offer virtual care to patients. Moreover, during the forced switch to virtual care, those who wanted evidence of success in high-frequency, low-touch care before agreeing to participate in the T1ME trial received it:

I worried that accountability wouldn’t be there as much, but I think that’s proven me wrong very much. Since COVID-19, I think people are more engaged even by phone.

#19

The pandemic provided an opportunity to explore both barriers and enablers of the current implementation of (low-touch) virtual care and distinguish them from those of high-frequency care. Like clinic staff, patients also had to get accustomed to virtual care visits. This included learning how to log on to and participate in virtual visits and read, interpret, upload, and share blood sugar data from pumps and continuous glucose monitors. Moreover, patients had to change their mindset and understand that the phone call was an actual visit. One CDE noted that her appointments were longer because patients were not as prepared for the virtual visit as they would be for an in-person appointment:

We tell [patients] that we’re going to be calling them for care. Next thing you know, “Well, wait a minute, my meter is up in my bedroom. Oh, wait a minute, my pills are in the kitchen.”

#20

However, most CDEs noted that as the pandemic progressed, patients acclimated to virtual care and began to enjoy it, which was a significant source of engagement for the CDEs:

Some people are saying, “So, do I have to come in the next time?” That’s kind of been the message. And almost everyone’s like, “I’m really fine if it’s this virtual again.” Very rarely do I get the question of, “When can I come in person?”

#33

### Implementation Plan

We identified the antecedent and key domains in our map (Figures 1-3) and linked them to the behavior change techniques shown to influence those specific domains. Additionally, during our member-checking process, we learned that clinic staff created a number of workarounds to offer a similar level of care virtually. We integrated these lessons into specific components of our implementation plan, such as the T1ME manual of operations. Thus, we created a comprehensive implementation and training plan targeting the key domains and lessons learned during the pandemic (Table 2).

**Table 2:** Implementation and training plan.

Component and its theoretical domains		Behavior change techniques	Actions
**Manual of operations**			
	Knowledge Belief about consequences Intention	Information about health consequences	Create 1-page summary of relevant literature supporting high-frequency, low-touch care
	Knowledge Skills Belief about capabilities	Problem solving Instruction on how to perform behavior	Plan how to resolve IT issues
	Belief about capabilities	Problem solving	Plan how to schedule and block off time for virtual visits FAQs^a^ (eg, what to do if patient misses virtual visit)
	Knowledge Skills Belief about capabilities	Instruction on how to perform behavior	Frequency and duration of virtual visits
**Training session**			
	Belief about capabilities	Demonstration of behavior	Create modeling session
	Belief about capabilities Skills	Demonstration of behavior Problem solving Rehearsal/practice	Create practice session processes (including with glitchy technology and common patient issues)
	Belief about capabilities	Problem solving	FAQs (eg, what to do if patient misses virtual visit)
**Monthly newsletters**			
	Reinforcement Social influences	Social reward	Highlight a CDE^b^ or site every month when they do something good in the trial
	Memory Attention and decision processes Environmental context and resources	Prompts/cues	Write feature piece on topic on virtual library
	Knowledge Belief about consequences Intention	Information on health consequences and social and environmental consequences (depending on what the patient story is about)	Write piece on patient stories
**Monthly meetings**			
	Reinforcement Social influences	Social reward	Compile success statistics
	Environmental context and resources Social influences	Practical and social support Problem solving	Create process for problem solving as a team
	Emotion	Reduce negative emotions	Create process for study team to gauge frustrations and come up with solutions
	Goal	Discrepancy between current behavior and goal	Create audit and feedback processes
**Data collection to share with CDEs and managers**			
	Belief in capabilities Belief in consequences	Information about social and environmental consequences Pros/cons comparative	Collect data on CDE workload
	Belief in capabilities Belief in consequences	Salience of consequences Information about social and environmental consequences Pros/cons comparative	Collect data on patient use

^a^FAQ: frequently asked question.

^b^CDE: certified diabetes educator.

## Discussion

### Principal Findings

It is estimated that only 20% of health research funding makes a public health impact [25]. This can be explained in part by the evidence-to-practice gap, which refers to the disconnect between the care that practitioners know is effective and that which is actually delivered [7]. To overcome this gap, implementation science approaches have been developed to understand the contextual factors of the setting in which the health care intervention is being implemented [6,8]. In this study, we took an implementation science approach to design and implement a virtual, high-frequency model of care intervention for type 1 diabetes clinics in Ontario, Canada, based on site-specific characteristics, semistructured interviews with clinic staff, and behavior change and implementation literature. Our interviews were completed before the COVID-19 pandemic; therefore, we completed a member-checking exercise during the pandemic to assess if our interview findings were still relevant within the context of predominantly virtual care.

Prior to the pandemic, health care providers in the T1D clinics we interviewed reported 2 main barriers in both the high frequency and low touch components. First, they shared the belief that this model of care would lead to an increased workload. Second, they felt that clinics did not have the necessary resources to implement the program successfully. However, during the pandemic when all clinics were utilizing virtual care, these clinics quickly developed strategies to overcome those barriers. Although the workload increased due to some clinic staff being redeployed to COVID-19 testing centers, those we spoke to felt that patients who needed care still received it. Additionally, some institutions invested in virtual care technology during this time, decreasing the barrier that was voiced prior to the pandemic regarding the lack of financial resources to obtain technology. These findings are encouraging, and they suggest that existing barriers to participating in virtual health care interventions can be overcome with the right support, such as technical training and resource allocation by the organization. However, these needs are not specific to diabetes clinics. Mohammed et al [26] showed that technical training and in-house organizational and administrative assistance were also important to primary care physicians and nurses in Ontario when using virtual care during the COVID-19 pandemic.

Our study also revealed important enablers to participating in a high-frequency, virtual health care intervention. During interviews that were conducted prior to the pandemic, participants noted an interest and intention among staff to deliver high-frequency, low-touch care and that continued engagement of staff would encourage the long-term success of studies such as the T1ME trial. Patient feedback was reported as being a great source of engagement for staff, and belief that the intervention would result in better patient outcomes was associated with an increased intention to participate in the trial. Similar to observations reported from health care providers across Canada during the pandemic, the CDEs we interviewed learned that patients were just as engaged in their care virtually as they were during the prepandemic period when most visits were conducted in person, and many patients wanted to continue virtual care as their primary means of follow up [27].

Lessons learned during the COVID-19 pandemic helped us update our implementation and training plan. Staff overcame some virtual care barriers (Figure 2) during the pandemic, and we used these lessons to make our implementation plan more robust. For example, we learned about common IT issues and how staff solved them, as well as about common patient issues and questions. We used these findings to update our manual of operations and training sessions. Moreover, we were able to collect examples of positive experiences between health care providers and patients using virtual care. These experiences will be used to increase the uptake of the T1ME trial by staff. Finally, now that clinic staff have become comfortable with virtual care, our team has focused more attention on tailoring our implementation plan to target factors surrounding a high-frequency care model. This includes dedicating more time to the modeling and practice portions of training sessions that offer guidance to CDEs on how to offer patient-centered care in shorter but more frequent touch points than are currently used.

### Strengths and Limitations

This study has a few notable strengths. First, we used a theory-based approach to create our interview guide. The TDF has mainly been used for implementation in health care contexts when understanding the behaviors of clinicians [19]. Therefore, we were able to ascertain significant implementation factors in this context. While other implementation theories have also been used to successfully implement complex interventions in health care, they come with limiting factors. For example, the Normalization Process Theory, which is centered around behavior rather than belief or intentions [28], has been criticized for focusing on the actions of health care providers rather than the experiences of the patients for whom the intervention is supposed to benefit [29]. Unlike our findings using TDF, Ross et al [10] found that the Normalization Process Theory did not account for the importance that diabetes health care providers placed on patient feedback; therefore, they were not able to include this factor in their implementation strategy. The TDF, however, does not come without critiques. For example, a strictly deductive analysis using the TDF will not allow non-TDF elements to be identified [30]. We overcame this limitation by using open coding when important issues were identified that did not clearly fit within an existing TDF domain. We also developed themes inductively within domains. Additionally, we will evaluate our implementation strategy, which will allow us to further refine our research plan to include any missing factors. Finally, our data collection prior to and during the pandemic facilitated the creation of a more robust implementation plan that can be applied to a variety of contexts. The feedback we obtained provides insights into what diabetes care will look like in the post–COVID-19 context so that we can adjust our research plan to meet these needs.

There are also a few limitations in our study to note. While we were able to interview diabetes staff in diverse clinics, all but 1 of our clinics were in urban settings. Therefore, the experiences of staff in these clinics may not reflect those of clinic staff in nonurban settings. Additionally, we were not able to reach staff from 3 clinics during the member-checking exercise, so we may have missed some important COVID-19–related barriers. Moreover, many members of our team are endocrinologists or research staff in diabetes clinics. Our prior experience and working relationships with some participants could be a potential bias in how we carried out the interviews and in our interpretation of the findings. Finally, end-user implementation factors were not assessed in this project. However, the T1ME trial team has conducted a separate project to understand the needs of people living with T1D regarding high-frequency, virtual care. Together, the results of both projects will give us a robust and comprehensive plan to implement the T1ME trial.

### Conclusion

For a complex health intervention to be successful, an implementation science approach is needed to understand contextual factors and identify levers that can support behavior change. Using site-specific characteristics, semistructured interviews with clinic staff, and behavior change and implementation literature, we developed a robust implementation and training plan to successfully implement a high-frequency, low-touch care model in diabetes clinics in Southern Ontario. Data were collected before and during the COVID-19 pandemic to enhance the effectiveness of our implementation strategy. An evaluation of our implementation plan in diabetes clinics in Toronto will allow us to create an improved iteration before applying it to other clinics in Ontario in the post–COVID-19 context.

## Abbreviations

ADP: Assistive Devices Program
BCT: behavior change technique
BCTTv1: Behavior Change Techniques Taxonomy Version 1
CDE: certified diabetes educator
EMR: electronic medical record
HbA1c: hemoglobin A1c
T1D: type 1 diabetes
T1ME: Type 1 Diabetes Virtual Self-Management Education and Support
TDF: Theoretical Domains Framework
